# An efficient numerical approach for singularly perturbed time delayed parabolic problems with two-parameters

**DOI:** 10.1186/s13104-024-06813-9

**Published:** 2024-06-06

**Authors:** Imiru Takele Daba, Wondewosen Gebeyaw Melesse, Fasika Wondimu Gelu, Guta Demisu Kebede

**Affiliations:** Mathematics, Dilla University, Dilla, 419 Ethiopia

**Keywords:** Non-standard finite difference method, Richardson extrapolation, Time delay, Two-parameter, 35A01, 65L10, 65L12, 65L20, 65L70

## Abstract

**Objectives:**

The main objective of this work is to design an efficient numerical scheme is proposed for solving singularly perturbed time delayed parabolic problems with two parameters.

**Results:**

The scheme is constructed via the implicit Euler and non-standard finite difference method to approximate the time and space derivatives, respectively. Besides, to enhance the accuracy and order of convergence of the method Richardson extrapolation technique is employed. Intensive numerical experimentation has been done on some model examples. Further, the layer behavior of the solutions is presented using graphs and observed to agree with the existing theories. Finally, error analysis of the scheme is done and observed that the proposed method is parameter uniform convergent with the order of convergence $\left( (\Delta t )^2+h^2\right)$

## Introduction

Many real-life problems in the fields of engineering and applied mathematics involve small positive parameter(s). Many researchers, scholars and engineers considered differential equations with one-parameter $\varepsilon (0<\varepsilon \ll 1)$, multiplying the highest derivative of the equation. This type of equation is one-parameter singularly perturbed differential equation. A number of research papers which deal with asymptotic, analytical and numerical solutions of such type of ordinary and partial differential equations can be found in the literature. Asymptotic and numerical solutions of two-parameter singularly perturbed differential equations are not studied extensively like their one-parameter counterpart.

It is a well-known fact that the presence of small parameters in the differential equations exhibit boundary and/or interior layers in the solution. O’Malley introduced singularly perturbed two-parameter problems and examined asymptotic expansion of their solutions [1–4]. In the subsequent years, several numerical methods were developed to improve the accuracy of the asymptotic methods proposed by O’Malley and his co-researchers [5]- [8]. From many researchers in the literature, author in [9] has proposed a robust fitted operator finite difference method for a two-parameter singular perturbation problem. Recently, quadratic B-spline collocation method for two-parameter singularly perturbed problem on exponentially graded mesh was proposed in [10].

The numerical solution of two-parametric singularly perturbed parabolic partial differential equations with non-smooth data were studied in [11–13]. Parameter-uniform numerical methods have been proposed by different researchers to solve two-parametric singularly perturbed parabolic partial differential equations with smooth data. To mention those methods, implicit Euler method in time on a uniform mesh and upwind finite difference method on Shishkin mesh [14], Rothe’s method for temporal discretization on a uniform mesh and finite element method for spatial discretization on a Shishkin mesh [15], an implicit Euler method in time and upwind finite difference method in space has been introduced using layer adapted equidistributed/moving meshes in [16], the classical implicit Euler method for time discretization and upwind scheme on the Shishkin-Bakhvalov mesh for spatial discretization was proposed in [17], an implicit Euler method for time discretization and a non-standard finite difference method on uniform mesh for spatial discretization was proposed in [18], the implicit Euler method for time stepping on a uniform mesh and a special hybrid monotone difference operator for spatial discretization on a specially designed piecewise uniform Shishkin mesh was considered in [19], an implicit Euler scheme on a uniform mesh in the temporal direction and the quadratic B-spline collocation scheme on an exponentially graded mesh in the spatial direction was constructed in [20], Crank-Nicolson scheme for the time derivative and cubic spline in tension for the spatial derivatives on a layer resolving non-uniform Bakhvalov-type mesh for a singularly perturbed two small parameters was presented in [22] to solve singularly perturbed two-parameter parabolic convection-diffusion–reaction problems. The authors in [40] have developed robust weak Galerkin finite element method for two parameter singularly perturbed parabolic problems on nonuniform meshes. The scholars in [43] have designed two hybrid computational algorithms to find approximate solutions for singularly perturbed parabolic convection-diffusion–reaction problems with two small parameters. All the aforementioned works for two-parameter singularly perturbed parabolic problems deals with initial-Dirichlet boundary conditions having no delay terms. However, the robust numerical solution of two-parameter singularly perturbed parabolic problems with delay terms are still limited.

Due to the wide application area of singularly perturbed problems with delay(s) have gained remarkable attention from researchers. For example, in population ecology, time delay represents the hatching period or duration of gestation; in genetic repression modeling, time delays play an important role in processes of transcription and translation as well as spatial diffusion of reactants and in control systems, delay terms account for the time delay in actuation and in information transmission and processing. Many other examples can be found in [34]. Also, the numerical treatment of such problems premeditated by many scholars. For instance, one-parameter singularly perturbed parabolic problems with time delay were studied in [23, 24, 36–38] and references therein. High-order finite difference technique for delay pseudo-parabolic equations is discussed in [41]. The authors [17] have established the finite difference scheme on Bakhvalov-type mesh for the singularly perturbed pseudo-parabolic problems with time-delay. Cimen and Amiraliyev [25] have suggested a uniform convergence method for singularly perturbed delay ordinary differential equation. Sumit et al. [26] have presented a robust numerical scheme using a hybrid monotone finite difference scheme on a rectangular mesh which is a product of uniform mesh in time and a layer-adapted Shishkin mesh in space for two-parametric singularly perturbed parabolic problems with time delay. Govindarao et al. [27] have developed a uniformly convergent computational method using the implicit Euler scheme for temporal discretization on a uniform mesh and the upwind difference scheme for the spatial discretization on the Shishkin type meshes (standard Shishkin mesh, Bakhvalov-Shishkin mesh) for singularly perturbed two parameter time delay parabolic problem. Kumar and Kumar [45] Have constructed numerical method using a hybrid monotone finite difference scheme on a rectangular mesh which is a product of uniform mesh in time and a layer-adapted Shishkin mesh in space for singularly perturbed two-parameter parabolic partial differential equations with time delay. The authors in [39] have suggested the high order difference approximation with Identity Expansions technique to construct a second order finite difference scheme and combine this with standard backward Euler difference scheme in a special way on a piecewise-uniform Shishkin mesh to solve coupled system of singularly perturbed first order ordinary differential equations. Multigrid techniques to solve the linear systems arising from finite difference discretization on Shishkin meshes of 2D singularly perturbed problems have presented in [44]

The schemes in [26] and [27] need apriori knowledge about the location and the width of the boundary layer(s) which might be difficult to understand for beginner researchers. Exponentially fitted difference (EFD) schemes have gained popularity as a powerful technique to solve boundary value problems. For instance, the authors in [31–33] have suggested different EFD schemes for singularly perturbed two-point boundary-value problems.

Motivated by the proceeding works, in this work, we constructed the non-standard difference method together with the Richardson extrapolation for two-parameter singularly perturbed parabolic problem with time delay. Let $\Omega _{s}^{N}=(0,1)$ and $\Omega _{t}^{M}=(0,T]$ be the space and time domain respectively, where *T* is fixed time. Then, the rectangular domain is the tensor product defined by $\Omega =\Omega _{s}^{N} \times \Omega _{t}^{M}$. Consider the following two-parameter singularly perturbed parabolic problem

1 $$\begin{aligned} {\left\{ \begin{array}{ll} \begin{aligned} {\mathcal {L}}_{\varepsilon ,\mu } z(s,t)&{}\equiv \varepsilon \frac{\partial ^{2} z(s,t)}{\partial s^{2}}+\mu a(s,t)\frac{\partial z(s,t)}{\partial s}-b(s,t)z(s,t)-\frac{\partial z(s,t)}{\partial t}\\ {} &{}=-c(s,t)z(s,t-\tau )+f(s,t), \;\; (s,t)\in \Omega , \end{aligned} \end{array}\right. } \end{aligned}$$

subject to initial condition

2 $$\begin{aligned} z(s,t)=\theta (s,t), \;\;\; (s,t)\in [0,1]\times [-\tau ,0], \end{aligned}$$

and boundary conditions

3 $$\begin{aligned} \begin{aligned} z(0,t)=q_0(t), \;\;\;\; 0\le t\le T,\\ z(1,t)=q_1(t), \;\;\;\; 0\le t\le T, \end{aligned} \end{aligned}$$

where $\varepsilon (0<\varepsilon \ll 1)$ and $\mu (0\le \mu \ll 1)$ are two small perturbation parameters and $\tau >0$ is delay parameter. For the existence and uniqueness of the solution, the functions *a*(*s*, *t*), *b*(*s*, *t*), *c*(*s*, *t*), *f*(*s*, *t*) $q_0(t), q_1(t)$ and $\theta (s,t)$ are sufficiently smooth and bounded with $a(s,t)\ge \alpha>0, b(s,t)\ge \beta >0$. The mathematical models related to two-parameter singularly perturbed problem of type (1) arises in transport phenomena in chemistry, biology, chemical reactor theory [4], lubrication theory [6] and dc motor theory [5] and flow through unsaturated porous media [8]. In two-parameter SPPs, the diffusion and convection terms are multiplied by the perturbation parameters.

Two-parameter singularly perturbed boundary value problems was first introduced by O’Malley, see [1–4]. He has shown that the layer behaviour for these problems depends not only on the parameters $\varepsilon$ and $\mu$, but significantly depends on the ratios of $\varepsilon$ to different powers of $\mu$. However, the ratio of $\mu ^2$ to $\varepsilon$ plays a significant role in the study of these problems. The particular cases, $\mu = 0$ in which the parabolic type layers each of width O$(\sqrt{\varepsilon })$ appear at both lateral boundary of the domain, and $\mu = 1$ in which an exponential layer of width O$(\varepsilon )$ appears near the left lateral boundary have been extensively considered analytically as well as numerically. For small $\varepsilon$ and $\mu$, the solution to the problem (1)–(3) exhibits boundary layers in the neighborhoods of $s=0$ and $s=1$.

**Notations:** Throughout this paper *N* and *M* denote the number of mesh points in *s* and *t* direction respectively. *C* denotes a generic positive constant independent of the singular perturbation parameters $\varepsilon , \mu$ and the mesh sizes.

## Analytical aspects of the problem

### Lemma 1

(Continuous minimum principle). Assume that $z(s,t)\in C^{(2,1)}({\bar{\Omega }})$ be sufficiently smooth function such that $z(s,t)\ge 0$ on $(s,t)\in [0,1]\times [-\tau ,0]$, $z(0,t)\ge 0$, $z(1,t)\ge 0$ on $0\le t\le T$, and ${\mathcal {L}}_{\varepsilon ,\mu }z(s,t)\le 0, (s,t)\in \Omega$. Then, $z(s,t)\ge 0$, $(s,t)\in \bar{\Omega }$.

### Proof

See [35] $$\square $$

The following Lemma proves the stability estimate to obtain unique solution.

### Lemma 2

(Uniform stability estimate) Let *z*(*s*, *t*) be the solution to the continuous problem (1)–(3), then it satisfies the bound

$$\begin{aligned} \Vert z \Vert _{{\bar{\Omega }}} \le \max \big \{ \vert \theta (s,t) \vert , \vert q_0(t) \vert , \vert q_1(t) \vert \big \}+\beta ^{-1}\Vert f\Vert , \end{aligned}$$

where $\left\| . \right\| _{\overline{\Omega }}$ is used to denote maximum norm given by $\left\| z\right\| _{\overline{\Omega }} =\max _{(s,t)\in \overline{\Omega }} |z(s,t)|.$

### Proof

See [35]. $$\square $$

## Description of the numerical method

We first discretized the time direction using an implicit Euler method with uniform step size $\Delta t$ which leads to a system of boundary value problem. Then, the discretization of space direction is made using the non-standard finite difference method.

### Time semi-discretization

We have two intervals $[-\tau , 0]$ and [0, *T*] on the time direction and we use the uniform mesh with time step $\Delta t$

$$\begin{aligned} \begin{array}{ll} \Omega ^{M}_{t}=\big \{t_{j}:t_{j}=j\Delta t, \; j=0,\cdots ,M, t_{M}=T, \Delta t=T/M\big \}, \\ \Omega ^{m}_{t}=\big \{t_{n}:t_{n}=n\Delta t, \; n=0,\cdots ,m, t_{m}=\tau , \Delta t=\tau /m \big \}, \end{array} \end{aligned}$$

where *M* is number of mesh points in t-direction in the interval [0, *T*] and *m* is the number of mesh points in $[-\tau , 0]$. The step length $\Delta t$ satisfies $m\Delta t = \tau$, where *m* is a positive integer and $t_{j} = j\Delta t, j \ge - m$. To discretize the time variable for Eq. (1), we use the implicit Euler method, which is given by

4 $$\begin{aligned} {\mathcal {L}}^M_{\varepsilon ,\mu }Z^{j+1}\equiv \varepsilon Z_{ss}(s,t_{j+1})+\mu a(s,t_{j+1})Z_s(s,t_{j+1})-d(s,t_{j+1})Z(s,t_{j+1})=g(s,t_{j+1}), \end{aligned}$$

subject to semi-discrete initial and boundary conditions

5 $$\begin{aligned} {\left\{ \begin{array}{ll} Z(s,t_{j+1})=\theta (s,t_{j+1}), &{} (s,t_{j+1})\in [0,1]\times [-\tau ,0],\\ Z(0,t_{j+1})=q_0(t_{j+1}), &{} 0\le t_{j+1}\le T,\\ Z(1,t_{j+1})=q_1(t_{j+1}), &{} 0\le t_{j+1}\le T, \end{array}\right. } \end{aligned}$$

where $d(s,t_{j+1})=b(s,t_{j+1})+\frac{1}{\Delta t}$ and $g(s,t_{j+1})=-c(s,t_{j+1})Z(s,t_{j+1-m})+f(s,t_{j+1})-\frac{Z(s,t_{j})}{\Delta t}$. By using the initial condition, we can evaluate the right-hand side as

$$\begin{aligned} g(s,t_{j+1})= {\left\{ \begin{array}{ll} \frac{Z(s,t_j)}{\Delta t}-c(s,t_{j+1})\theta (s,t_{j+1-m})+f(s,t_{j+1}), &{} j=0,1,...,m,\\ \frac{Z(s,t_j)}{\Delta t}-c(s,t_{j+1})Z(s,t_{j+1-m})+f(s,t_{j+1}), &{} j=m+1,...,M. \end{array}\right. } \end{aligned}$$

The local truncation error of the time semi-discretization is given by $e_{j+1}=z(s,t_{j+1})-Z(s,t_{j+1})$, where $Z(s,t_{j+1})$ is the solution of the following boundary value problem

6 $$\varepsilon {Z_{ss}}\left( {s,{t_{j + 1}}} \right) + \mu a\left( {s,{t_{j + 1}}} \right){Z_s}\left( {s,{t_{j + 1}}} \right) - d\left( {s,{t_{j + 1}}} \right)Z\left( {s,{t_{j + 1}}} \right) = g\left( {s,{t_{j + 1}}} \right),$$

with the boundary conditions

7 $$\begin{aligned} {\left\{ \begin{array}{ll} Z(0,t_{j+1})=q_0(t_{j+1}), &{} 0\le t_{j+1}\le T,\\ Z(1,t_{j+1})=q_1(t_{j+1}), &{} 0\le t_{j+1}\le T. \end{array}\right. } \end{aligned}$$

Now, we state the bounds for the errors in the local and global as follows.

#### Lemma 3

(Local error estimate) If

$$\begin{aligned} \bigg |\frac{\partial ^k Z(s,t)}{\partial s^k} \bigg |\le C, \hspace{1cm} (s,t)\in \bar{\Omega }, ~~ 0\le k\le 2, \end{aligned}$$

the local error estimate of the time discretization is given by

$$\begin{aligned} \Vert e_{j+1} \Vert _\infty \le C\Delta t^2, \;\;\; 1\le j \le M. \end{aligned}$$

#### Proof

One can find the proof of lemma in [27].$\square$

The global error is the measure of the contribution of the local error estimate at each time step and is given by $$ E_{j}=z(s,t_j)-Z(s,t_j).$$

#### Lemma 4

Under Lemma (3), the global error estimate at $t_{j}$ is given by

$$\begin{aligned} \Vert E_{j}\Vert _{\infty } \le C\Delta t, ~~~ j\le T/\Delta t. \end{aligned}$$

We conclude that time semi-discretization is first-order uniformly convergent. The $\varepsilon -$convergence analysis of the numerical scheme which we propose requires that we use bounds on the solution and its derivatives. The solutions of the characteristic equation for time semi-discrete problem

$$\begin{aligned} \varepsilon r^2(s,t_j)+\mu a(s,t_{j+1})r(s,t_{j+1})-(b(s,t_{j+1})+1/\Delta t)=0 \end{aligned}$$

are $r_0(s)<0$ and $r_1(s)>0$ which are used to describe the boundary layers at $s=0$ and $s=1$, respectively. The quantities $\mu _0$ and $\mu _1$ are defined as $\mu _0=-\max \limits _{[0,1]}r_0(s)$ and $\mu _1=\max \limits _{[0,1]}r_1(s)$.

#### Remark 1

The situations of two external layers are characterized by $\mu ^2\ll 1$ or $\mu ^2/\varepsilon \rightarrow 0$ as $\varepsilon \rightarrow 0$, which implies that $\mu _0 \approx \mu _1 \approx \min \limits _{[0,1]} \sqrt{\frac{(b(s,t_{j+1})+1/\Delta t)}{\varepsilon }}$ and we have boundary layers at $s=0$ and $s=1$. The boundary layer at $s=0$ is encountered in the case when $\varepsilon \ll \mu ^2$ as $\mu \rightarrow 0$. In this case, $\mu _1\approx 0$ and $\mu _0 \approx \min \limits _{[0,1]} \frac{\mu a(s,t_j)}{\varepsilon }$.

The next theorem, gives the $\varepsilon$-uniform bounds for the derivatives of the solution *u* with respect to *x*, needs to study the uniform convergence at spatial discretization.

#### Theorem 1

Up to a certain order *k* that depends on the smoothness of of the functions *a*(*s*, *t*), *b*(*s*, *t*), *f*(*s*, *t*) and for any real constant $p\in (0,1)$, we have the following bound

$$\begin{aligned} \bigg \Vert \frac{\partial ^{k} z(s)}{\partial s^k} \bigg \Vert _{\bar{\Omega }} \le C\big (1+\mu _0^ke^{-p\mu _0s}+\mu _1^ke^{-p\mu _1(1-s)}\big ), \hspace{0.5cm} 0\le k \le q. \end{aligned}$$

#### Proof

For the details of the proof, see [15]. $\square$

### Spatial discretization

In this section, we discretize the spatial variable of (4) using the nonstandard finite difference method of Mickens [29] as discussed below. On the spatial domain [0, 1], we introduce the equidistant meshes with uniform mesh length *h* such that $s_{0}=0, s_{i}=ih,i=1(1)N-1,s_{N}=1,h=\frac{1}{N}$, where *h* is the step size and *N* is the number of mesh points in the spatial direction. The spatial Discretization of (4) yields

8 $$\begin{aligned} \begin{array}{c} \varepsilon \frac{\partial ^{2} Z(s_i,t_{j+1})}{\partial s^{2}}+\mu a(s_i,t_{j+1})\frac{\partial Z(s_i,t_{j+1})}{\partial s}-b(s_i,t_{j+1})Z(s_i,t_{j+1})-\frac{Z(s_i,t_{j+1}-Z(s_i,t_{j}}{\Delta t}\\ =-c(s_i,t_{j+1})Z(s_i,t_{j+1-m})+f(s_i,t_{j+1}). \end{array} \end{aligned}$$

We use the notation $Z(s_i,t_{j+1})\equiv Z_i$ as the approximation of $z(s_i,t_{j+1})\equiv z_i$ for the sake of simplicity. From the theory of non-standard finite difference method, we can discretize (8) in space to obtain the discrete problem in the form

9 $$\begin{aligned} \begin{array}{ll} {\mathcal {L}}^{N,M}_{\varepsilon ,\mu }Z_i\equiv \varepsilon \bigg (\frac{ Z_{i-1}-2Z_i+Z_{i+1}}{\gamma ^2_i}\bigg )+\mu a_i\bigg (\frac{Z_{i+1}-Z_i}{h}\bigg ) -d_iZ_i=g_i. \end{array} \end{aligned}$$

where $d_i=b_i+\frac{1}{\Delta t}$ and $g_i=-c_iZ_i^{-m}+f_i-\frac{Z_i}{\Delta t}$ for $i=1,...,N-1, \;\; j=0,...,M.$ According to Mickens [28, 29], the concept of sub-equations is the major tool to derive the denominator function for the differential equation. From (9), we take the homogeneous form of the constant coefficient sub-equation

10 $$\begin{aligned} \varepsilon \bigg (\frac{ Z_{i-1}-2Z_i+Z_{i+1}}{\gamma ^2_i}\bigg )+\delta \bigg (\frac{Z_{i+1}-Z_i}{h}\bigg ) -\eta Z_i=0, \end{aligned}$$

where $\delta =\mu a_i$ and $\eta =d_i=b_i+\frac{1}{\Delta t}$. Equation (10) has two linearly independent analytical solutions, namely, $\exp (\lambda _{1} x)$ and $\exp (\lambda _{2} x)$, where

11 $$\begin{aligned} \lambda _{1,2}=\frac{-\delta \pm \sqrt{\delta ^{2}-4\varepsilon \eta }}{2\varepsilon }. \end{aligned}$$

Following Micken’s, we construct the second-order difference equation for (10) as follows

12 $$\begin{aligned} {\left| \begin{array}{ccc} Z_{i-1}&{}Z_{1},_{i-1}&{}Z_{2},_{i-1}\\ Z_{i}&{}Z_{1},_{i}&{}Z_{2},_{i}\\ Z_{i+1}&{}Z_{1},_{i+1}&{}Z_{2},_{i+1} \end{array} \right| } =\left| \begin{array}{ccc} Z_{i-1}&{}\exp (\lambda _{1} x_{i-1})&{}\exp (\lambda _{2} x_{i-1})\\ Z_{i}&{}\exp (\lambda _{1} x_{i})&{}\exp (\lambda _{2} x_{i})\\ Z_{i+1}&{}\exp (\lambda _{1} x_{i+1})&{}\exp (\lambda _{2} x_{i+1}) \end{array}\right| =0. \end{aligned}$$

Simplifying the determinant in (12), we get

13 $$\begin{aligned} -\exp (\frac{\delta h}{2\varepsilon })Z_{i+1}+2\cosh \bigg (\frac{h\sqrt{\delta ^{2}+4\varepsilon \eta }}{2\varepsilon }\bigg )Z_{i}-\exp (\frac{-\delta h}{2\varepsilon })Z_{i-1}=0, \end{aligned}$$

which is the exact scheme for (10) in the sense that (13) has the same general solution

14 $$\begin{aligned} Z_{i}=C_{1}\exp (\lambda _{1} x_{i})+C_{2}\exp (\lambda _{2} x_{i}) \end{aligned}$$

as (10). It is noted that to construct the non-standard finite difference method, we need to find a suitable denominator function which replaces $h^2$. To this end, the extraction of the denominator function from (13) is not straightforward. However, the fact that the layer behaviors of the solution of problem (1) and that of the problem (10) in the case when $\eta \equiv 0$ are similar [9]. Thus, for the latter case, that is, $\eta \equiv 0$, we have the exact scheme of the form

15 $$\begin{aligned} -\exp (\frac{\delta h}{2\varepsilon })Z_{i+1}+\bigg (\exp (\frac{\delta h}{2\varepsilon })+\exp (\frac{-\delta h}{2\varepsilon })\bigg )Z_{i}-\exp (\frac{-\delta h}{2\varepsilon })Z_{i-1}=0, \end{aligned}$$

from hyperbolic identity. Multiplying both sides of equation (15) by $\exp (\frac{\delta h}{2\varepsilon })$, we have

$$\begin{aligned} \exp (\frac{\delta h}{\varepsilon })Z_{i+1}-\bigg (\exp (\frac{\delta h}{\varepsilon })+1\bigg )Z_{i}+Z_{i-1}=0, \end{aligned}$$

Adding the term $(Z_{i+1}+Z_{i})$ and subtracting it and after some manipulations, we have

16 $${Z_{i + 1}} - 2{Z_i} + {Z_{i - 1}} + \left( {\exp (\frac{{\delta h}}{\varepsilon }) - 1} \right)\left( {{Z_{i + 1}} - {Z_i}} \right) = 0.$$

From (16), we have

17 $$\begin{aligned} \varepsilon \frac{ Z_{i-1}-2Z_i+Z_{i+1}}{\gamma ^{2}}+\delta \frac{Z_{i+1}-Z_i}{h}=0, \end{aligned}$$

where the denominator function is given by

18 $$\begin{aligned} \gamma ^{2}(h,\varepsilon ,\mu )\equiv \gamma ^{2}=\frac{h\varepsilon }{\mu a}\bigg (\exp (\frac{\mu a h}{\varepsilon })-1\bigg ). \end{aligned}$$

Adopting the denominator function for the variable coefficient, we can write as

19 $$\begin{aligned} \gamma _i^{2}(h,\varepsilon ,\mu )\equiv \gamma _i^{2}=\frac{h\varepsilon }{\mu a_i}\bigg (\exp (\frac{\mu a_i h}{\varepsilon })-1\bigg ). \end{aligned}$$

Thus, we get the following fully discrete problem

20 $$\begin{aligned} \begin{array}{l} \varepsilon \frac{ Z_{i-1}^{j+1}-2Z_i^{j+1}+Z_{i+1}^{j+1}}{\gamma _i^2}+\mu a_i^{j+1}\bigg (\frac{Z_{i+1}^{j+1}-Z_i^{j+1}}{h}\bigg )-b_i^{j+1}Z_i^{j+1}-\frac{ Z_i^{j+1}-Z_i^{j}}{\Delta t}\\ =-c_i^{j+1}Z_i^{j+1-m}+f_i^{j+1}, \end{array} \end{aligned}$$

with the following discrete initial and boundary conditions

21 $$\begin{aligned} {\left\{ \begin{array}{ll} Z_i^{-j}=\theta (s_i,-t^{j}), &{} i=1,\cdots ,N-1, \;\; j=0,\cdots , m,\\ Z_0^{j+1}=q_0(t_{j+1}), &{} t_{j+1}\in [0,T],\\ Z_N^{j+1}=q_1(t_{j+1}), &{} t_{j+1}\in [0,T], \end{array}\right. } \end{aligned}$$

where the denominator function is given as

$$\begin{aligned} \gamma _i^{2}(h,\varepsilon ,\mu )\equiv \gamma _i^{2}=\frac{h\varepsilon }{\mu a_i^{j+1}}\bigg (\exp (\frac{\mu a_i^{j+1} h}{\varepsilon })-1\bigg ). \end{aligned}$$

The discrete scheme in (20) and the discrete conditions in (21) can be written in matrix form as

22 $$\begin{aligned} WZ=G, \;\;\; i=1,2,...,N-1, \;\;\; j=0,...,M, \end{aligned}$$

where *Z* and *G* are column vectors of $N-1$ and the matrix *W* is a tri-diagonal matrix of $(N-1)\times (N-1)$. The entries of the coefficient matrix *W* are given by

23 $$\begin{aligned} {\left\{ \begin{array}{ll} W_{i},_{i-1}=\frac{\varepsilon }{\gamma ^2_{i}}, &{} i=1,...,N-2,\\ W_{i},_{i}=-(\frac{2\varepsilon }{\gamma ^2_i}+\frac{\mu a_i^{j+1}}{h}+\frac{1}{\Delta t}+b_i^{j+1}), &{} i=1,...,N-1,\\ W_{i},_{i+1}=\frac{\varepsilon }{\gamma ^2_{i}}+\frac{\mu a_i^{j+1}}{h}, &{} i=1,...,N-1. \end{array}\right. } \end{aligned}$$

The entries of column vectors *G* and *Z* are given as follows

24 $$\begin{aligned} {\left\{ \begin{array}{ll} G_0^{j+1}=q_0(t_{j+1}),\\ G_i^{j+1}=-c_i^{j+1}Z_i^{j+1-m}+f_i^{j+1}-\frac{Z_i^{j}}{\Delta t}, &{} i=1(1)N-1,\\ G_N^{j+1}=q_1(t_{j+1}),\\ Z=[Z_0,Z_1,\cdots ,Z_N]^{T}. \end{array}\right. } \end{aligned}$$

Next, we prove some useful attributes the discrete scheme in (22). The discrete operator ${\mathcal {L}}_{\varepsilon ,\mu }^{N,M}$ defined in (9) satisfies the following discrete minimum principle.

#### Theorem 2

Assume that ${\mathcal {L}}_{\varepsilon ,\mu }^{N,M}$ be discrete operator given in (9) and $\Pi _i^j$ be any mesh function that satisfies the initial condition $\Pi _i^{-j}\ge 0, 1\le i \le N-1, \;\; 0\le j \le m$ and boundary conditions $\Pi _0^j\ge 0, \; \Pi _N^j\ge 0$, $0\le j \le M$. If ${\mathcal {L}}_{\varepsilon ,\mu }^{N,M}\Pi _i^j\le 0$ for all $(i,j)\in \Omega ^{N,M}$, then $\Pi _i^j\ge 0$ in $\bar{\Omega }^{N,M}.$

#### Proof

Let *s* and *p* be indices such that $\Pi _{s}^{p}= \min \limits _{{\forall (i,j)}}\Pi _i^j$ for $\Pi _i^j\in \bar{\Omega }^{N,M}$. Assume that $\Pi _{s}^{p}<0$. Then, it is easy to see that $(s,p) \in \{1,\cdots ,N-1\}\times \{1,\cdots ,M\}$, because otherwise $\Pi _{s}^{p}\ge 0$. It follows that $\Pi _{s+1}^p-\Pi _s^p\ge 0$ and $\Pi _s^p-\Pi _{s-1}^p\le 0$. Therefore, now

$$\begin{aligned} \begin{aligned} {\mathcal {L}}_{\varepsilon ,\mu }^{N,M}\Pi _{s}^p&=\frac{\varepsilon }{\gamma ^2_{s}}(\Pi _{s+1}^{p+1}-2\Pi _{s}^{p+1}+\Pi _{s-1}^{p+1})+\frac{\mu a_s^{p+1}}{h}(\Pi _{s+1}^{p+1}-\Pi _s^{p+1})-d_s^{p+1}\Pi _s^{p+1},\\&=\frac{\varepsilon }{\gamma ^2_s}[(\Pi _{s+1}^{p+1}-\Pi _s^{p+1})-(\Pi _s^{p+1}-\Pi _{s-1}^{p+1})]+\frac{\mu a_s^{p+1}}{h}(\Pi _{s+1}^{p+1}-\Pi _s^{p+1})-d_s^{p+1}\Pi _s^{p+1},\\&\ge 0, \end{aligned} \end{aligned}$$

which is a contradiction and thus, the assumption $\Pi _s^{p+1}<0, ~\forall (s,p)$ is wrong. Thus, $\Pi _s^{p+1}>0$ implies that $\Pi _i^j\ge 0$ in $\bar{\Omega }^{N,M}.$ $\square$

Using this discrete minimum principle, we now show that the present method also satisfies the uniform stability estimate given in the following lemma.

#### Lemma 5

The discrete operator ${\mathcal {L}}_{\varepsilon ,\mu }^{N,M}$ is uniformly stable, in the sense that if $P_i^{j+1}$ is any mesh function such that $P_0^{j+1}=P_N^{j+1}=0$, then

$$\begin{aligned} \vert P_i^{j+1} \vert \le \beta ^{-1} \max \limits _{1\le i \le N-1}\vert {\mathcal {L}}_{\varepsilon ,\mu }^{N,M}P_i^{j+1}\vert , \;\;\; \text {for}\;\; 0<i<N. \end{aligned}$$

#### Proof

Let define the two barrier functions $(\Psi ^{\pm })_i^j$ by $(\Psi ^{\pm })_i^j=Y \pm P_i^{j+1},$ where

$$\begin{aligned} =\beta ^{-1} \max \limits _{1\le i \le N-1}\vert {\mathcal {L}}_{\varepsilon ,\mu }^{N,M}P_i^{j+1}\vert . \end{aligned}$$

We have $(\Psi ^{\pm })_0^{j+1}=Y \pm P_0^{j+1}=Y \pm q_0(t_{j+1})\ge 0$, and $(\Psi ^{\pm })_N^{j+1}=Y \pm P_N^{j+1}=Y \pm q_1(t_{j+1})\ge 0.$

On the discretized domain $1\le i \le N-1$, we have

$$\begin{aligned} {\mathcal {L}}_{\varepsilon ,\mu }^{N,M}(\Psi ^{\pm })_i^{j+1}\equiv -\frac{(b_i^{j+1}+1/\Delta t)}{\beta }\max \limits _{1\le i \le N-1}\vert {\mathcal {L}}_{\varepsilon ,\mu }^{N,M}P_i^{j+1}\vert \pm {\mathcal {L}}_{\varepsilon ,\mu }^{N,M}P_i^{j+1}. \end{aligned}$$

Using the fact that where $0<\beta \le b_i^{j+1}<(b_i^{j+1}+1/\Delta t)$, we have ${\mathcal {L}}_{\varepsilon ,\mu }^{N,M}(\Psi ^{\pm })_i^{j+1}\le 0$. By the discrete minimum principle in Theorem (2), we obtain $(\Psi ^{\pm })_i^{j+1}\ge 0,\; 0\le i \le N.$ $\square$

### Convergence analysis

We use the following lemma to prove the uniform convergence analysis of the discrete problem (9).

#### Lemma 6

For all positive integers *k* on a fixed mesh, we have

$$\begin{aligned} \lim \limits _{\varepsilon \rightarrow \ 0} \max \limits _{1<i<N-1}\frac{\exp (\frac{-Cx_{i}}{\sqrt{\varepsilon }})}{\varepsilon ^{\frac{k}{2}}}=0 \;\;\; \text{ and } \;\;\; \lim \limits _{\varepsilon \rightarrow \ 0} \max \limits _{1<i<N-1}\frac{\exp (\frac{-C(1-x_{i})}{\sqrt{\varepsilon }})}{\varepsilon ^{\frac{k}{2}}}=0, \end{aligned}$$

where $$x_{i}=ih, h=\frac{1}{N}, \hspace{0.2cm} \forall i=1,...,N-1.$$

#### Proof

For the proof, see [30]. $\square$

The following analysis concerns the space variable *x*. The local truncation error in the spatial variable of the proposed method is given by

$$\begin{aligned} \begin{aligned} {\mathcal {L}}_{\varepsilon ,\mu }^{N,M}(Z_i^{j+1}-z_i^{j+1})&=({\mathcal {L}}_{\varepsilon ,\mu } -{\mathcal {L}}_{\varepsilon ,\mu }^{N,M})z_i^{j+1}, \\&={\mathcal {L}}_{\varepsilon ,\mu }z_i^{j+1}-{\mathcal {L}}_{\varepsilon ,\mu }^{N,M}z_i^{j+1},\\&=\varepsilon \left( z^{\prime \prime }\right) _i^{j+1}+\mu a_i^{j+1} \left( z^{\prime }\right) _i^{j+1}-b_i^{j+1}z_i^{j+1}\\&-\bigg [\varepsilon \bigg (\frac{z_{i+1}^{j+1}-2z_i^{j+1}+z_{i-1}^{j+1}}{\gamma _i^2}\bigg )+\mu a_i^{j+1}\bigg (\frac{z_{i+1}^{j+1}-z_i^{j+1}}{h}\bigg )-b_i^{j+1}z_i^{j+1}\bigg ]. \end{aligned} \end{aligned}$$

Simplifying the above expression, we obtain

25 $$\begin{aligned} \begin{aligned} {\mathcal {L}}_{\varepsilon ,\mu }^{N,M}(Z_i^{j+1}-z_i^{j+1})&= \varepsilon \left( z^{\prime \prime }\right) _i^{j+1}+\mu a_i^{j+1}\left( z^{\prime }\right) _i^{j+1}\\ {}&-\varepsilon \bigg (\frac{z_{i+1}^{j+1}-2z_i^{j+1}+z_{i-1}^{j+1}}{\gamma _i^2}\bigg )-\mu a_i^{j+1}\bigg (\frac{z_{i+1}^{j+1}-z_i^{j+1}}{h}\bigg ). \end{aligned} \end{aligned}$$

Taylor series expansions of the terms $z_{i}^{j+1}, z_{i+1}^{j+1}$ and $z_{i-1}^{j+1}$ on space direction are given as following

$$\begin{aligned} \begin{aligned} z_{i+1}^{j+1}&=z_i(t)+h\left( z^{\prime }\right) _i^{j+1}+\frac{h^2}{2}\left( z^{\prime \prime }\right) _i^{j+1}+\frac{h^3}{6}\left( z^{\prime \prime \prime }\right) _i^{j+1}+\frac{h^4}{24}\left( z^{\prime \prime \prime \prime }\right) _i^{j+1}+\cdots \\ z_{i-1}^{j+1}&=z_i^{j+1}-h\left( z^{\prime }\right) _i^{j+1}+\frac{h^2}{2}\left( z^{\prime \prime }\right) _i^{j+1}-\frac{h^3}{6}\left( z^{\prime \prime \prime }\right) _i^{j+1}+\frac{h^4}{24}\left( z^{\prime \prime \prime \prime }\right) _i^{j+1}+\cdots . \end{aligned} \end{aligned}$$

Adding the first two equations in the above Taylor series expansion, we deduce the following

26 $$\begin{aligned} z_{i+1}^{j+1}-2z_i^{j+1}+z_{i-1}^{j+1}=h^2\left( z^{\prime \prime }\right) _i^{j+1}+\frac{h^4}{12}\left( z^{\prime \prime \prime \prime }\right) _i^{j+1}+\cdots . \end{aligned}$$

From the first Taylor series expansion, we have

27 $$\begin{aligned} z_{i+1}^{j+1}-z_i^{j+1}=h\left( z^{\prime }\right) _i^{j+1}+\frac{h^2}{2}\left( z^{\prime \prime }\right) _i^{j+1}+\frac{h^3}{6}\left( z^{\prime \prime \prime }\right) _i^{j+1}+\cdots . \end{aligned}$$

Substituting equations (26)-(27) in their respective positions into (25) and rearranging, we obtain

28 $$\begin{aligned} \begin{aligned} {\mathcal {L}}_{\varepsilon ,\mu }^{N,M}(Z_i^{j+1}-z_i^{j+1})&= \varepsilon \left( z^{\prime \prime }\right) _i^{j+1}+\mu a_i^{j+1}\left( z^{\prime }\right) _i^{j+1}- \frac{\varepsilon }{\gamma _i^2} \bigg (h^2\left( z^{\prime \prime }\right) _i^{j+1}+\frac{h^4}{12}\left( z^{\prime \prime \prime \prime }\right) _i^{j+1}\bigg )\\ {}&-\frac{\mu a_i^{j+1}}{h}\bigg (h\left( z^{\prime }\right) _i^{j+1}+\frac{h^2}{2}\left( z^{\prime \prime }\right) _i^{j+1}+\frac{h^3}{6}\left( z^{\prime \prime \prime }\right) _i^{j+1}\bigg ). \end{aligned} \end{aligned}$$

Simplifying (28), we obtain

29 $$\begin{aligned} \begin{aligned} {\mathcal {L}}_{\varepsilon ,\mu }^{N,M}(Z_i^{j+1}-z_i^{j+1})&= \varepsilon \left( z^{\prime \prime }\right) _i^{j+1} -\frac{\varepsilon }{\gamma _i^2} \bigg (h^2\left( z^{\prime \prime }\right) _i^{j+1}+\frac{h^4}{12}\left( z^{\prime \prime \prime \prime }\right) _i^{j+1}\bigg )-\bigg (\frac{\mu a_i(t)}{2}\left( z^{\prime \prime }\right) _i^{j+1}\bigg ) h\\bigg(\frac{\mu a_i^{j+1}}{6}\left( z^{\prime \prime \prime }\right) _i^{j+1}\bigg )h^2. \end{aligned} \end{aligned}$$

Using a truncated Taylor series expansion of the denominator function [18]

30 $$\begin{aligned} \frac{1}{\gamma _i^2}=\frac{1}{h^2}-\frac{\mu a_i^{j+1}}{2\varepsilon h}+\frac{\mu ^{2} \left( a_i^{j+1}\right) ^{2}}{12\varepsilon ^{2} } \end{aligned}$$

in (30) gives

31 $$\begin{aligned} \begin{aligned} {\mathcal {L}}_{\varepsilon ,\mu }^{N,M}(Z_i^{j+1}-z_i^{j+1})&=\bigg (\mu a_i^{j+1}\left( z^{\prime \prime }\right) _i^{j+1}\bigg )h-\bigg (\frac{(\mu a_i^{j+1})^2}{12\varepsilon }\left( z^{\prime \prime }\right) _i^{j+1}+\frac{\mu a_i(^{j+1}}{6}\left( z^{\prime \prime \prime \prime }\right) _i^{j+1}\bigg )h^2. \end{aligned} \end{aligned}$$

Applying the relation $h>h^2$ in (31) and using the bounds on derivatives in Theorem (1) together with Lemma (6) and using the fact that as $\varepsilon \rightarrow 0$, both the terms $\mu _0^k\exp (-p\mu _0s_i)$ and $\mu _1^k\exp (-p\mu _1(1-s_i))\rightarrow 0$ for all $k\in \{0,1,2,\cdots ,\}$. Thus, the discrete problem satisfies the following bound

$$\begin{aligned} \vert {\mathcal {L}}_{\varepsilon ,\mu }^{N,M}(Z_i^{j+1}-z_i^{j+1})\vert \le Ch. \end{aligned}$$

where *C* is constant independent of the perturbation parameters and mesh sizes. Invoking the uniform stability in Lemma (5), we obtain the result

$$\begin{aligned} \vert Z_i^{j+1}-z_i^{j+1}\vert \le Ch. \end{aligned}$$

Therefore, main convergence of the fully discretized scheme is given in the following theorem.

#### Theorem 3

Let $z_i^{j+1}\in C^{4,2}(\bar{\Omega })$ be the solution to problem in (1)-(3)) and $Z_i^{j+1}$ be the solution to discrete problem in (20) with its discrete boundary conditions in (21). Then, the overall error bound satisfies

32 $$\begin{aligned} \max \limits _{0\le i\le N; 0\le j\le M} \vert Z_i^{j+1}-z(s_i,t_{j+1})\vert \le C(h+\Delta t). \end{aligned}$$

From the above theorem, we deduce that the developed method is first-order convergent, independent of the parameters $\varepsilon$ and $\mu$ both in space and time directions.

Next, we use Richardson extrapolation to boost the accuracy and rate of convergence for the proposed method.

### Richardson extrapolation

Richardson extrapolation is a technique used to accelerate the accuracy and rate of convergence of the proposed method. From (32), we have

33 $$\begin{aligned} \vert Z_i^{j+1}-z(s_i,t_{j+1})\vert \le C(h+\Delta t), \end{aligned}$$

where $z(s_i,t_{j+1})$ and $Z_i^{j+1}$ are exact and approximate solutions, respectively. Assume $\Omega ^{N,M}\subset \Omega ^{2N,2M}$ where $\Omega ^{N,M}$ is the mesh obtained from the mesh intervals *h* and $\Delta t$ and $\Omega ^{2N,2M}$ is the mesh obtained by bisecting the mesh intervals *h* and $\Delta t$. Denoting the numerical solution obtained with the mesh points $\Omega ^{2N,2M}$ by $\tilde{Z}_i^{j+1}$. From (32), it is clear that for the mesh $(s_i,t_{j+1})\in \Omega ^{N,M}$

34 $$\begin{aligned} Z_i^{j+1}-z(s_i,t_{j+1}) \le C(h+\Delta t)+R^{N,M}, \end{aligned}$$

As ${\tilde{s}}_{i+1}-{\tilde{s}}_{i}={\tilde{h}}=\frac{h}{2}$ for ${\tilde{s}}_i\in \Omega ^{2N}$ and ${\tilde{t}}_{j+1}-{\tilde{t}}_{j}=\tilde{\Delta }t=\frac{\Delta t}{2}$ for ${\tilde{t}}_{j+1}\in \Omega ^{2\,M}.$ For the mesh $({\tilde{s}}_i,{\tilde{t}}_{j+1})\in \Omega ^{2N,2M}$, we have

35 $$\begin{aligned} \tilde{Z}_i^{j+1}-z(s_i,t_{j+1}) \le C(\frac{h}{2}+\frac{\Delta t}{2})+R^{2N,2M}, \end{aligned}$$

where $R^{N,M}$ and $R^{2N,2\,M}$ are the remainder terms with the truncation error of $O(h^2+\Delta t^2)$. Combining the inequalities in (34) and (35) gives us with $z(s_i,t_{j+1})-(2\tilde{Z}_i^{j+1}-z(s_i,t_{j+1}))\le C(h^2+\Delta t^2)$, which yields that

36 $$\begin{aligned} (Z_i^{j+1})^{extr}=2\tilde{Z}_i^{j+1}-Z_i^{j+1}, \end{aligned}$$

is also an extrapolated numerical solution. Therefore, we have the error bound for extrapolated solution summarized in the theorem as follows.

#### Theorem 4

Let $z_i^{j+1}$ be the solution to the continuous problem (1) and (2) and $(Z_i^{j+1})^{extr}$ be the extrapolated solution. Then, the new error bound takes the form

$$\begin{aligned} \sup \limits _{0<\varepsilon \le 1} \ \max \limits _{0\le i\le N; 0\le j\le M} \vert (Z_i^{j+1})^{extr}-z(s_i,t_{j+1})\vert \le C((\Delta t)^2+h^2). \end{aligned}$$

#### Proof

The proof is given in [21]. $\square$

Therefore, using Richardson extrapolation, first-order uniformly convergent method is changed into second-order uniformly convergent. Thus, the proposed method is second-order convergent.

## Test examples, numerical computations and discussions

In this section, we carry out numerical experiments to corroborate the performance of the proposed method with the theoretical results discussed in the previous sections. Since the exact solution for the Examples (1) and (2) are not available, we use the double mesh principle to calculate maximum absolute errors, for each $(\varepsilon ,\mu )$, using the following formula

$$\begin{aligned} e_{\varepsilon ,\mu }^{N,M}=\max \limits _{0\le i\le N;t\in [0,T]}\big \vert Z^{N,M}(s_i,t_j)-Z^{2N,2M}(s_i,t_j)\big \vert , \end{aligned}$$

before extrapolation (BE) and after extrapolation (AE), we use the formula

$$\begin{aligned} (e_{\varepsilon ,\mu }^{N,M})^{extr}=\max \limits _{0\le i\le N;t\in [0,T]}\big \vert (Z^{N,M})^{extr}(s_i,t_j)-(Z^{2N,2M})^{extr}(s_i,t_j)\big \vert , \end{aligned}$$

where $Z^{N,M}(s_i,t_j)$ is the numerical solution with (*N*, *M*) mesh points and $Z^{2N,2M}(s_i,t_j)$ is the numerical solution at the finer mesh with (2*N*, 2*M*) mesh points before extrapolation (BE). The numerical solutions after extrapolation (AE) are $(Z^{N,M})^{extr}(s_i,t_j)$ using the mesh points (*N*, *M*) with mesh sizes *h* and $\Delta t$ and $(Z^{2N,2M})^{extr}(s_i,t_j)$ using the mesh points (2*N*, 2*M*) with mesh sizes $\frac{h}{2}$ and $\frac{\Delta t}{2}$. The $(\varepsilon ,\mu )$-uniform errors before and after extrapolation were calculated using the following formulas, respectively

$$\begin{aligned} e^{N,M}=\max \limits _{\varepsilon ,\mu }e_{\varepsilon ,\mu }^{N,M}\;\;\; \text{ and } \;\;\; (e^{N,M})^{extr}=\max \limits _{\varepsilon ,\mu }(e_{\varepsilon ,\mu }^{N,M})^{extr}. \end{aligned}$$

Furthermore, we compute the numerical rate of convergence before and after extrapolation with the following formulas, respectively

$$\begin{aligned} \rho _{\varepsilon ,\mu }^{N,M}=\log _{2}\bigg (\frac{e_{\varepsilon ,\mu }^{N,M}}{e_{\varepsilon ,\mu }^{2N, 2M}}\bigg ) \;\;\; \text{ and } \;\;\; (\rho _{\varepsilon ,\mu }^{N,M})^{extr}=\log _{2}\bigg (\frac{(e_{\varepsilon ,\mu }^{N,M})^{extr}}{(e_{\varepsilon ,\mu }^{2N, 2M})^{extr}}\bigg ). \end{aligned}$$

The $(\varepsilon ,\mu )$-uniform rate of convergence before and after extrapolation were calculated using the following formulas, respectively

$$\begin{aligned} \rho ^{N,M}=\max \limits _{\varepsilon ,\mu }\rho _{\varepsilon ,\mu }^{N,M}\;\;\; \text{ and } \;\;\; \rho _{extr}^{N,M}=\max \limits _{\varepsilon ,\mu }(\rho _{\varepsilon ,\mu }^{N,M})^{extr}. \end{aligned}$$

### Example 1

Consider singularly perturbed two-parameter parabolic problem [26, 27]

$$\begin{aligned} {\left\{ \begin{array}{ll} \begin{aligned} &{}\varepsilon \frac{\partial ^{2} z}{\partial s^{2}}+\mu (1+s)\frac{\partial z}{\partial s}-z(s,t)-\frac{\partial z}{\partial t} =-z(s,t-1)+16s^2(1-s^2), \hspace{0.5cm} (s,t)\in (0,1)\times (0,2],\\ {} &{}z(s,t)=0, \hspace{2cm} (s,t)\in (0,1)\times (-1,0],\\ &{}z(0,t)=0, \hspace{2cm} z(1,t)=0, \hspace{2cm} t\in (0,2]. \end{aligned} \end{array}\right. } \end{aligned}$$

### Example 2

Consider singularly perturbed two-parameter parabolic problem [26, 27]

$$\begin{aligned} {\left\{ \begin{array}{ll} \begin{aligned} &{}\varepsilon \frac{\partial ^{2} z}{\partial s^{2}}+\mu (1+s(1-s)+t^2)\frac{\partial z}{\partial s}-(1+5st)z(s,t)-\frac{\partial z}{\partial t}=-z(s,t-1)+s(1-s)(e^t-1),\\ {} &{} (s,t)\in (0,1)\times (0,2],\;\;\;\\ &{}z(s,t)=0, \hspace{2cm} (s,t)\in (0,1)\times (-1,0],\\ {} &{}z(0,t)=0, \hspace{2cm} z(1,t)=0, \hspace{2cm} t\in (0,2]. \end{aligned} \end{array}\right. } \end{aligned}$$

**Table 1:** Comparison of $e^{N,M}_{\varepsilon ,\mu }, \; (e_{\varepsilon ,\mu }^{N,M})^{extr}$ and $\rho ^{N,M}_{\varepsilon ,\mu }$ for fixed $\mu =10^{-4}$ and varying $\varepsilon$ for Example (1) with $e^{N,M}_{\varepsilon ,\mu }$ in [27] using Shishkin (S-) mesh and Bakhvalov-Shishkin (BS-) mesh

$\varepsilon \downarrow$	$N=32$	64	128	256	512	1024
	$M=32$	64	128	256	512	1024
AE						
$10^{-4}$	1.1796e-04	3.0426e-05	8.5400e-06	2.3807e-06	6.1817e-07	1.5593e-07
	1.9549	1.8330	1.8429	1.9453	1.9871	–
$10^{-6}$	1.1675e-04	3.0012e-05	7.6144e-06	1.9185e-06	4.8156e-07	1.2601e-07
	1.9598	1.9787	1.9888	1.9942	1.9342	–
$10^{-8}$	1.1759e-04	3.0329e-05	7.7060e-06	1.9420e-06	4.8724e-07	1.1815e-07
	1.9550	1.9766	1.9884	1.9948	2.0440	–
$10^{-10}$	1.1759e-04	3.0329e-05	7.7060e-06	1.9420e-06	4.8746e-07	1.2211e-07
	1.9550	1.9766	1.9884	1.9884	1.9971	–
BE						
$10^{-4}$	5.5308e-03	2.8187e-03	1.4231e-03	7.1501e-04	3.5838e-04	1.7941e-04
	0.9725	0.9860	0.9930	0.9965	0.9982	–
$10^{-6}$	5.5355e-03	2.8202e-03	1.4235e-03	7.1511e-04	3.5840e-04	1.7942e-04
	0.9729	0.9864	0.9932	0.9966	0.9982	–
$10^{-8}$	5.5364e-03	2.8214e-03	1.4244e-03	7.1564e-04	3.5869e-04	1.7956e-04
	0.9725	0.9861	0.9930	0.9965	0.9983	–
$10^{-10}$	5.5364e-03	2.8214e-03	1.4244e-03	7.1564e-04	3.5869e-04	1.7956e-04
	0.9725	0.9861	0.9930	0.9930	0.9983	–
S-mesh [27]						
$10^{-4}$	2.8183e-03	1.4253e-03	7.1638e-04	3.5904e-04	1.7973e-04	–
	0.9835	0.9925	0.9965	0.9983	0.9992	
$10^{-6}$	2.8274e-03	1.4272e-03	7.1717e-04	3.5952e-04	1.7999e-04	–
	0.9863	0.9927	0.9962	0.9981	0.9991	
$10^{-8}$	2.8287e-03	1.4280e-03	7.1748e-04	3.5961e-04	1.8002e-04	–
	0.9860	0.9930	0.9965	0.9982	0.9991	
$10^{-10}$	2.8286e-03	1.4280e-03	7.1747e-04	3.5960e-04	1.8002e-04	–
	0.9861	0.9930	0.9965	0.9982	0.9991	
BS-mesh [27]						
$10^{-4}$	1.9776e-03	9.9679e-04	5.0025e-04	2.5059e-04	1.2544e-04	–
	0.9892	0.9946	0.9973	0.9986	0.9993	
$10^{-6}$	1.9789e-03	9.9691e-04	5.0033e-04	2.5064e-04	1.2544e-04	–
	0.9891	0.9945	0.9973	0.9986	0.9993	
$10^{-8}$	1.9789e-03	9.9691e-04	5.0033e-04	2.5064e-04	1.2544e-04	–
	0.9891	0.9945	0.9973	0.9981	0.9993	
$10^{-10}$	1.9789e-03	9.9691e-04	5.0033e-04	2.5064e-04	1.2544e-04	–
	0.9891	0.9945	0.9973	0.9986	0.9993

**Table 2:** Comparison of $(e^{N,M}),\; (e^{N,M})^{extr}, \; \rho ^{N,M}$, and $(\rho ^{N,M})^{extr}$ for Example (1) with [26] using $\mu =10^{-3}$

	$N=32$	64	128	256	512	1024
	$M=8$	16	32	64	128	256
AE						
$e^{N,M}$	1.9905e-02	1.0684e-02	5.5440e-03	2.8252e-03	1.4263e-03	7.1660e-04
$\rho ^{N,M}$	0.8977	0.9465	0.9726	0.9861	0.9930	–
In [26]						
$e^{N,M}$	4.3705e-02	1.6704e-02	7.3802e-03	3.7406e-03	1.8967e-03	9.5511e-04
$\rho ^{N,M}$	1.3876	1.1785	0.9803	0.9797	0.9898	–

**Table 3:** Comparison of $(e^{N,M}),\; (e^{N,M})^{extr}, \; \rho ^{N,M}$, and $(\rho ^{N,M})^{extr}$ for Example (1) with [26] using $\mu =10^{-9}$

	$N=32$	64	128	256	512	1024
	$M=8$	16	32	64	128	256
AE						
$(e^{N,M})^{extr}$	1.7326e-03	5.0411e-04	1.3530e-04	3.5089e-05	8.9410e-06	2.2556e-06
$(\rho ^{N,M})^{extr}$	1.7811	1.8976	1.9471	1.9725	1.9869	–
BE						
$e^{N,M}$	1.9856e-02	1.0659e-02	5.5315e-03	2.8189e-03	1.4231e-03	7.1502e-04
$\rho ^{N,M}$	0.8975	0.9463	0.9725	0.9861	0.9930	–
In [26]						
$e^{N,M}$	4.3817e-02	1.6750e-02	7.4019e-03	3.7490e-03	1.9008e-03	9.5719e-04
$\rho ^{N,M}$	1.3873	1.1781	0.9813	0.9799	0.9898	–

**Table 4:** $e^{N,M}_{\varepsilon ,\mu }, \; (e_{\varepsilon ,\mu }^{N,M})^{extr}$, $\rho ^{N,M}_{\varepsilon ,\mu }$ and $\left( \rho ^{N,M}_{\varepsilon ,\mu }\right) ^{extr}$ for $\mu =10^{-6}$ and varying $\varepsilon$ for Example (2)

$\varepsilon \downarrow$	$N=32$	64	128	256	512	1024
	$M=32$	64	128	256	512	1024
AE						
$10^{-4}$	2.2712e-05	1.0185e-05	6.9936e-06	2.1124e-06	5.6980e-07	1.4413e-07
	1.1570	0.5423	1.7272	1.8904	1.9831	–
$10^{-6}$	2.2714e-05	6.6067e-06	1.7924e-06	4.6775e-07	1.1961e-07	4.2250e-08
	1.7816	1.8820	1.9381	1.9674	1.5013	–
$10^{-8}$	2.2714e-05	6.6067e-06	1.7924e-06	4.6775e-07	1.1962e-07	3.0249e-08
	1.7816	1.8820	1.9381	1.9673	1.9835	–
$10^{-10}$	2.2714e-05	6.6067e-06	1.7924e-06	4.6775e-07	1.1961e-07	3.0249e-08
	1.7816	1.8820	1.9381	1.9674	1.9834	–
BE						
$10^{-4}$	1.1968e-03	6.0516e-04	3.0384e-04	1.5225e-04	7.6202e-05	3.8121e-05
	0.9838	0.9940	0.9969	0.9985	0.9992	–
$10^{-6}$	1.2014e-03	6.0665e-04	3.0454e-04	1.5260e-04	7.6376e-05	3.8206e-05
	0.9858	0.9942	0.9969	0.9986	0.9993	–
$10^{-8}$	1.2014e-03	6.0663e-04	3.0453e-04	1.5260e-04	7.6376e-05	3.8207e-05
	0.9858	0.9942	0.9968	0.9986	0.9993	–
$10^{-10}$	1.2014e-03	6.0663e-04	3.0453e-04	1.5260e-04	7.6376e-05	3.8205e-05
	0.9858	0.9942	0.9968	0.9986	0.9993	–

**Table 5:** Comparison of $(e^{N,M}),\; (e^{N,M})^{extr}, \; \rho ^{N,M}$, and $(\rho ^{N,M})^{extr}$ for Example (2) with [26] using using $\mu =10^{-3}$

	$N=32$	64	128	256	512	1024
	$M=8$	16	32	64	128	256
AE						
$(e^{N,M})^{extr}$	2.0431e-04	6.9968e-05	2.7254e-05	7.9009e-06	3.5478e-06	4.4385e-06
$(\rho ^{N,M})^{extr}$	1.5460	1.3602	1.7864	1.7355	0.9035	–
BE						
$e^{N,M}$	4.5677e-03	2.3673e-03	1.2057e-03	6.0797e-04	3.0526e-04	1.5295e-04
$\rho ^{N,M}$	0.9482	0.9734	0.9878	0.9940	0.9970	–
In [26]						
$e^{N,M}$	1.1161e-02	5.1087e-03	2.4749e-03	1.2214e-03	6.0706e-04	3.0264e-04
$\rho ^{N,M}$	1.1274	1.0455	1.0188	1.0086	1.0042	–

**Table 6:** Comparison of $(e^{N,M}),\; (e^{N,M})^{extr}, \; \rho ^{N,M}$, and $(\rho ^{N,M})^{extr}$ for Example (2) with [26] using $\mu =10^{-9}$

	$N=32$	64	128	256	512	1024
	$M=8$	16	32	64	128	256
AE						
$e^{N,M}$	4.5780e-03	2.3685e-03	1.2040e-03	6.0686e-04	3.0462e-04	1.5261e-04
$\rho ^{N,M}$	0.9507	0.9761	0.9884	0.9944	0.9972	–
In [26]						
$e^{N,M}$	1.1100e-02	5.0838e-03	2.4640e-03	1.2162e-03	6.0457e-04	3.0142e-04
$\rho ^{N,M}$	1.1265	1.0449	1.0185	1.0084	1.0041	–

**Table 7:** Comparison of $e^{N,M}_{\varepsilon ,\mu }, \; (e_{\varepsilon ,\mu }^{N,M})^{extr}$, $\rho ^{N,M}_{\varepsilon ,\mu }$, $(e^{N,M})^{extr}$ and $(\rho ^{N,M})^{extr}$ for $\epsilon =10^{-4}$ and varying $\mu$ for Example (1) with [27]

$\mu \downarrow$	$N=32$	64	128	256	512	1024
	$M=32$	64	128	256	512	1024
AE						
$10^{-4}$	1.1796e-04	3.0426e-05	8.5400e-06	2.3807e-06	6.1817e-07	1.5593e-07
	1.9549	1.8330	1.8429	1.9453	1.9871	–
$10^{-6}$	1.1798e-04	3.0430e-05	8.5160e-06	2.3728e-06	6.1533e-07	1.5530e-07
	1.9550	1.8372	1.8436	1.9472	1.9863	
$10^{-8}$	1.1798e-04	3.0430e-05	8.5160e-06	2.3728e-06	6.1533e-07	1.5530e-07
	1.9550	1.8372	1.8436	1.9472	1.9863	
$10^{-10}$	1.1798e-04	3.0430e-05	8.5160e-06	2.3728e-06	6.1533e-07	1.5530e-07
	1.9550	1.8372	1.8436	1.9472	1.9863	
$(e^{N,M})^{extr}$	1.1798e-04	3.0430e-05	8.5160e-06	2.3728e-06	6.1533e-07	1.5530e-07
$(\rho ^{N,M})^{extr}$	1.9550	1.8372	1.8436	1.9472	1.9863	–
BE						
$10^{-4}$	5.5308e-03	2.8187e-03	1.4231e-03	7.1501e-04	3.5838e-04	1.7941e-04
	0.9725	0.9860	0.9930	0.9965	0.9982	–
$10^{-6}$	5.5307e-03	2.8187e-03	1.4231e-03	7.1501e-04	3.5838e-04	1.7941e-04
	0.97243	0.98599	0.99300	0.99647	0.99823	
$10^{-8}$	5.5308e-03	2.8187e-03	1.4231e-03	7.1501e-04	3.5838e-04	1.7941e-04
	0.97243	0.98599	0.99300	0.99647	0.99823	
$10^{-10}$	5.5308e-03	2.8187e-03	1.4231e-03	7.1501e-04	3.5838e-04	1.7941e-04
	0.97243	0.98599	0.99300	0.99647	0.99823	
$e^{N,M}$	5.5308e-03	2.8187e-03	1.4231e-03	7.1501e-04	3.5838e-04	1.7941e-04
$\rho ^{N,M}$	0.97243	0.98599	0.99300	0.99647	0.99823	
S-mesh [27]						
$e^{N,M}$	2.8183e-3	1.4253e-3	7.1638e-4	3.5904e-4	1.7973e-4	–
$\rho ^{N,M}$	0.9835	0.9925	0.9965	0.9983	0.9992	–
B-mesh [27]						
$e^{N,M}$	1.9653e-3	9.9001e-4	4.9686e-4	2.4891e-4	1.2531e-4	–
$\rho ^{N,M}$	0.9892	0.9946	0.9973	0.9986	0.9901	–

**Table 8:** Comparison of $e^{N,M}_{\varepsilon ,\mu }, \; (e_{\varepsilon ,\mu }^{N,M})^{extr}$ and $\rho ^{N,M}_{\varepsilon ,\mu }$ for $\epsilon =10^{-4}$ and varying $\mu$ for Example (2)

$\mu \downarrow$	$N=32$	64	128	256	512
	$M=32$	64	128	256	512
AE					
$10^{-4}$	3.8567e-05	9.5725e-06	2.3842e-06	6.6341e-07	1.7709e-07
	2.0104	2.0054	1.8455	1.9054	–
$10^{-6}$	3.8555e-05	9.5698e-06	2.3835e-06	5.9476e-07	1.4855e-07
	2.0104	2.0054	2.0027	2.0014	–
$10^{-8}$	3.8555e-05	9.5698e-06	2.3836e-06	5.9476e-07	1.4853e-07
	2.0104	2.0053	2.0028	2.0016	–
$10^{-10}$	3.8555e-05	9.5698e-06	2.3836e-06	5.9476e-07	1.4853e-07
	2.0104	2.0053	2.0028	2.0016	–
BE					
$10^{-4}$	7.5295e-03	3.7840e-03	1.8968e-03	9.4960e-04	4.7510e-04
	0.99264	0.99634	0.99818	0.99909	–
$10^{-6}$	7.5295e-03	3.7840e-03	1.8968e-03	9.4960e-04	4.7510e-04
	0.99264	0.99634	0.99818	0.99909	–
$10^{-8}$	7.5295e-03	3.7840e-03	1.8968e-03	9.4960e-04	4.7510e-04
	0.99264	0.99634	0.99818	0.99909	–
$10^{-10}$	7.5295e-03	3.7840e-03	1.8968e-03	9.4960e-04	4.7510e-04
	0.99264	0.99634	0.99818	0.99909	–

**Fig. 1:**
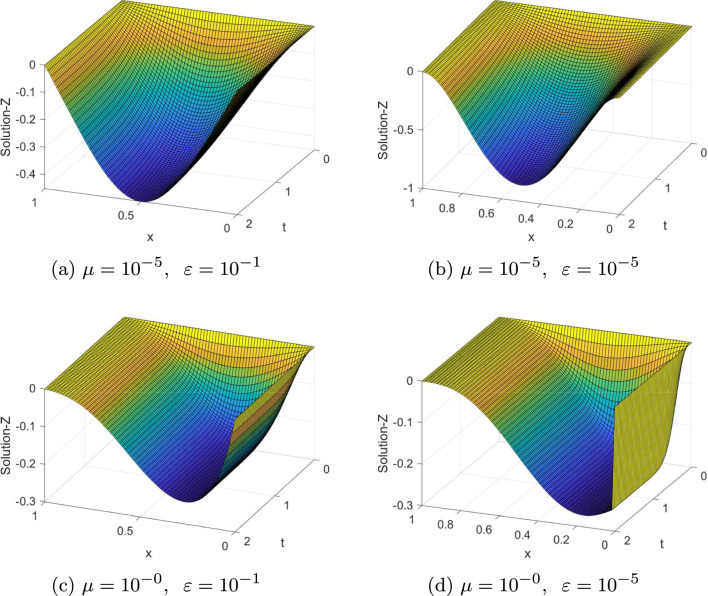
3-D view of the numerical solution profiles for Example 1 at $N=64=M$

**Fig. 2:**
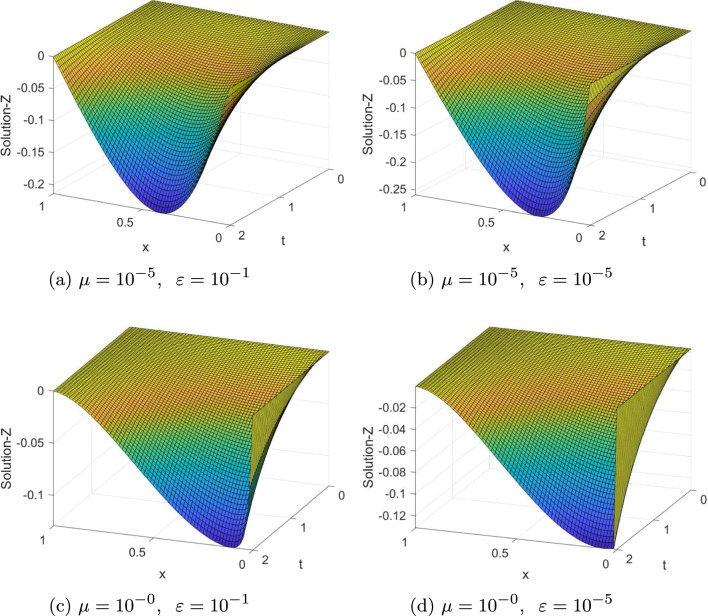
3-D view of the numerical solution profiles for Example (2) at $N=64=M$

**Fig. 3:**
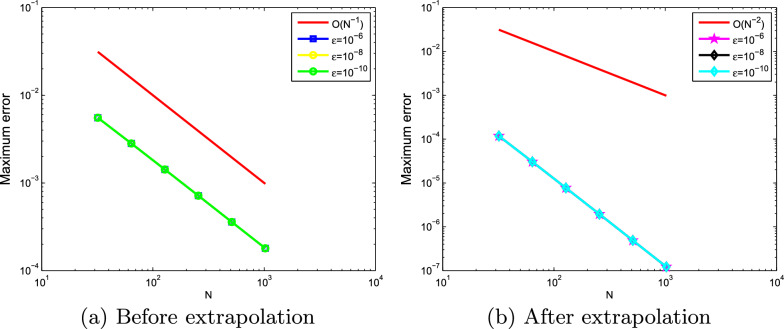
Loglog plot of the maximum point-wise errors at $\mu =10^{-4}$ for Example (1)

**Fig. 4:**
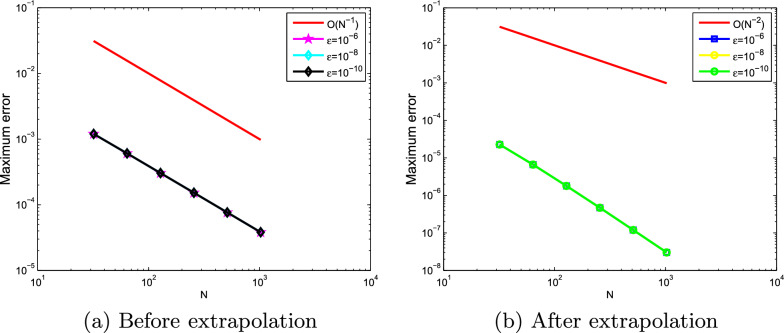
Loglog plot of the maximum point-wise errors at $\mu =10^{-6}$ for Example (2)

The computed $(e_{\varepsilon , \mu }^{N,M}),\; (e_{\varepsilon , \mu }^{N,M})^{extr}, \;(e^{N,M}),\; (e^{N,M})^{extr}, \; \rho _{\varepsilon , \mu }^{N,M}$, $(\rho _{\varepsilon , \mu }^{N,M})^{extr}\; \rho ^{N,M}$, and $(\rho ^{N,M})^{extr}$ for Examples (1) and (2) are tabulated in Tables 1, 2, 3, 4, 5, 6, 7 and 8 for different values of $\varepsilon , \mu$ and mesh points. From these tables, one can observe that the results obtained after extrapolation provides more accurate results obtained before extrapolation and results in [26, 27]. From the table of values, we deduce that when the mesh points increases the maximum absolute errors decreases. Numerical simulation for Examples (1 and 2) are displayed in Figs. (1 and 2), respectively. From these figures, we observe that as $(\varepsilon ,\mu )$ goes very small a twin boundary layers are created at $s=0$ and $s=1$. For a visual understanding of the theoretical order of convergence graphically, the maximum absolute errors for Examples (1) and (2) are plotted using log-log scale in Figs. (3 and 4), respectively.

## Conclusion

In this study, a robust numerical method for the two-parametric singularly perturbed time-delayed parabolic problem on a uniform mesh is presented. The problem is discretized by an implicit Euler method in the time variable and the non-standard finite difference method in the space variable. The method is analyzed for parameter uniform convergence. To boost the accuracy, the Richardson extrapolation technique has been applied. The numerical solutions displayed in the Tables show that the present method is parameter uniform convergence of second-order and it agrees with the theoretical order of convergence. The performance of the proposed method is examined by comparing the results with those of previous studies. It has been found that the proposed scheme provides more accurate and stable results. To substantiate the suitability of the proposed method, graphs have been plotted for the two examples by taking different values of the parameters $\varepsilon$ and $\mu$. The drawback of the proposed method is that difficult to apply to higher-order singular perturbation problems. The proposed method is easy to implement and, with a little modification, can easily be extended to nonlinear, discontinuous data, and other families of the problem under consideration.

## Data Availability

Not applicable.
